# Oral administration of cysteine peptides attenuates UV-B-induced skin erythema and pigmentation in humans

**DOI:** 10.1038/s41598-024-73447-z

**Published:** 2024-09-27

**Authors:** Ayako Sakuma, Yumiko Kai, Yoshimitsu Yamasaki, Tomoya Tanaka, Takanobu Sakurai

**Affiliations:** 1 Research and Development Division, Mitsubishi Corporation Life Sciences Limited, Tokyo, 100-0006 Japan; 2 Maruishilabo Corporation, Osaka, 531-0071 Japan; 3 Osaka Nishiumeda Clinic, Osaka, 530-0001 Japan; 4 HUMA R&D CORP, Tokyo, 108-0023 Japan

**Keywords:** Cysteine peptides, Glutathione, γ-glutamylcysteine, Cysteinylglycine, Ultraviolet, UV, MED, Erythema, Pigmentation, Health care, Medical research

## Abstract

The oral administration of antioxidants may suppress UV-B-induced skin damage. HITHION YH-15, the extract of Torula yeast (*Cyberlindnera jadinii*), is rich in cysteine-containing peptides such as reduced and oxidized glutathione (GSH and GSSG), γ-glutamylcysteine (γ-Glu-Cys), and cysteinylglycine (Cys-Gly). These four constituents are termed cysteine peptides. In this study, we investigated the protective effects of cysteine peptides against UV-B in a randomized, placebo-controlled, double-blind, parallel-group study. A total of 90 healthy males and females aged 30–59 years were enrolled and randomized into two groups of 45 individuals each (cysteine peptides (48 mg/day) and placebo). Changes in UV-B-induced erythema and pigmentation were compared between groups after 5 weeks of test food intake. The minimal erythema dose (MED) significantly increased (*p = 0.019) in the cysteine peptides group compared to that in the placebo group, indicating suppression of UV-B-induced erythema. ΔL* value significantly increased (***p < 0.0001) in the cysteine peptides group compared to that in the placebo, indicating pigmentation suppression. We demonstrated that oral administration of cysteine peptides suppresses UV-B-induced erythema and pigmentation through multiple mechanisms. Thus, cysteine peptides may find use as nutricosmetics for maintaining skin health and well-being.

UMIN Clinical Trials Registry ID: UMIN 000050157.

## Introduction

Excessive exposure of the skin to ultraviolet (UV) radiation causes increased oxidative stress and numerous skin disorders, including skin cancer. The UV radiation reaching the Earth’s surface is approximately 90% UV-A and 10% UV-B, with the latter being 1,000–10,000 fold more carcinogenic than UV-A^1^. UV-B causes acute reactions such as erythema and pigmentation through the production of reactive oxygen species (ROS) and the absorption of UV-B by cellular biomolecules^2^. In addition to physical strategies to avoid UV radiation, such as parasols and sunscreens, the administration of antioxidants, such as astaxanthin, vitamin E, carotenoids, and polyphenols, can suppress UV-B-induced skin damage, as shown recently^3–5^. Glutathione (GSH), a master antioxidant, is widely recognized as a skin-whitening agent^6^. Its estimated mechanisms include its antioxidant and anti-melanogenesis effects^6–8^. In previous human clinical studies using GSH alone, efficacy was observed at doses of 250 and 500 mg^6–8^. However, to the best of our knowledge, no previous studies have reported on its protective effects against UV-B-induced skin deterioration in humans.

HITHION YH-15, the extract of Torula yeast (*Cyberlindnera jadinii*) produced by Mitsubishi Corporation Life Sciences Limited, is rich in cysteine-containing peptides such as reduced and oxidized glutathione (GSH and GSSG), and its constituents: γ-glutamylcysteine (γ-Glu-Cys) and cysteinylglycine (Cys-Gly). These four constituents of GSH are termed cysteine peptides^9,10^, are expected to exhibit antioxidant activity. GSH is a potent antioxidant present in the body that eliminates ROS to protect mammalian cells from oxidative damage^11^. In our previous clinical study with HITHION YH-15, its skin-brightening effect, without UV irradiation, was observed at 12 weeks at a low dose (48 mg/day as cysteine peptides)^12^. Since cysteine peptides refer to a group of peptides that constitute GSH, we hypothesized that a low dose of cysteine peptides may also have a protective effect against UV-induced skin deterioration such as erythema and pigmentation. To test this hypothesis, the present study was conducted on healthy men and women aged 30–59 years. This is the first study to report the protective effect of oral supplementation of cysteine peptides, namely, GSH, GSSG, γ-Glu-Cys and Cys-Gly, against UV-B-induced erythema at 4 weeks and pigmentation at 5 weeks in humans.

## Methods

### Study design

A randomized, placebo-controlled, double-blind, parallel-group study was conducted on 90 healthy Japanese males and females aged 30–59 years to assess the effect of oral supplementation with cysteine peptides for 5 weeks on UV-B-induced erythema and pigmentation. Detailed study schedules are presented in Table 1. The minimal erythema dose (MED), the primary outcome, was determined before and after 4 weeks of the intervention. Pigmentation, as the secondary outcome, was determined by measuring skin brightness and melanin synthesis using ΔL* and Δmelanin index, respectively, (Δ = 1.5 MED irradiated area—non-irradiated area) 7 days after irradiation with 1.5 MED, based on the baseline MED, before and after 5 weeks of the intervention. This study was approved by the Youga Allergy Clinic Clinical Research Ethics Review Committee on 20/1/2023 (IRB: 21000023), and registered in the UMIN Clinical Trials Registry on 27/1/2023 (Identifier: UMIN000050157). Both the research institute and the contract research organization complied with the “Declaration of Helsinki”, the “Ethical Guidelines for Life Sciences and Medical Research Involving Human Subjects” and the “Act on the Protection of Personal Information” in conducting the study.

**Table 1:**
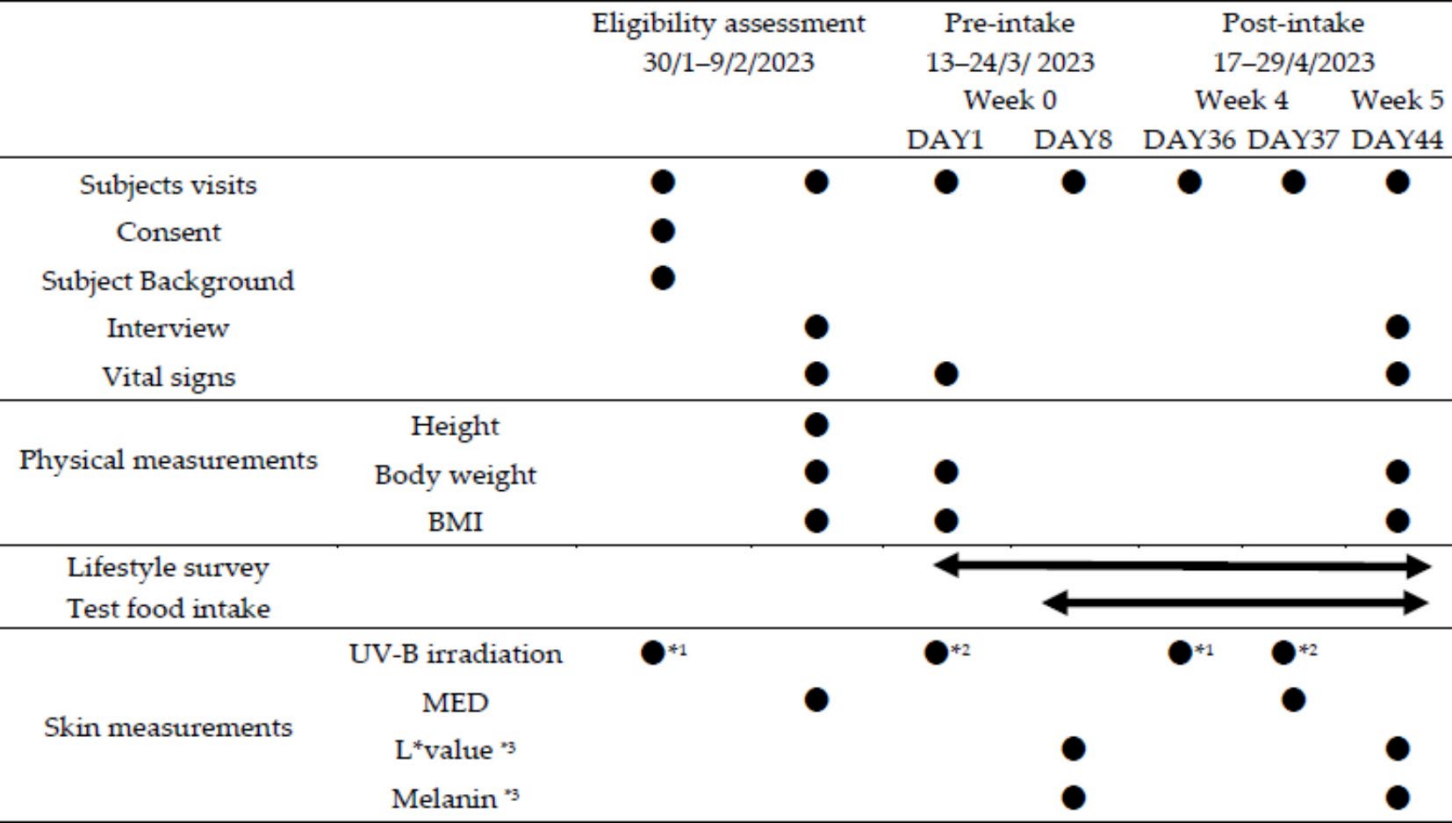
Study design.

*1：UV-B irradiation patterns: 11.57, 13.30, 15.30, 17.56, 20.22, 23.28, 26.73, 30.72, 35.38, 40.70, 46.82, and 53.87(mJ/cm2).

*2：UV-B irradiation of 1.5 times the value determined by MED at the eligibility assessment.

*3：Values were measured 7 days after 1.5 MED irradiation at two points, one at the site irradiated with 1.5 MED and the other at the non-irradiated site.

### Sample size, randomization, and blinding

A previous study using 48 mg cysteine peptides per day reported a skin-brightening effect with 15 participants per group^12^, and another study using 500 mg GSH per day with 30 participants per group investigated its effect on melanin index^7^. However, neither the effect of cysteine peptides nor that of GSH on MED have been investigated to date, which was the primary outcome of this study. To estimate the sample size required for the experiment, changes in MED and standard deviation (SD) in previous studies with other antioxidants^5,13^ were also taken into account. The required sample size was calculated using the following parameters: the difference of MED = 5 mJ/cm^2^, SD = 8 mJ/cm^2^, α = 0.05, statistical power (1-β) = 0.80, resulting in a sample size of 41 participants per group, which led us to settle on a sample size of 45 participants per group. The test food allocation manager assigned subjects to two groups, a cysteine peptides group and a placebo group, by stratified randomization after incorporation and before the start of the intervention. The allocation factors included age, sex, skin type, and MED. The software used was R Version 3.5.2 (R Core Team, Vienna, Austria). The study food allocation manager strictly maintained the test food allocation list until key opening, and blinding was maintained for all parties except for the study food allocation manager.

### Participants

Participants aged 30–59 years from Japan were enrolled in this study. Participants received an explanation of the objectives and details of this study and provided written informed consent to participate on 30/1/2023 and 1, 3, 6, and 8/2/2023. Participants were enrolled as per the following inclusion criteria: (1) Japanese men and women between 30 and 59 years of age at the time of written consent; (2) those with Fitzpatrick skin phototype II or III (skin phototype in which the skin turns reddish and then darkens when exposed to the sun for 30–45 min during the spring and summer); and (3) those who had been informed of the purpose and content of the study, were competent to give informed consent, voluntarily agreed to participate in the study based on a thorough understanding of the purpose and content of the study, and provided written informed consent to participate in the study. Participants were excluded based on the following criteria: (1) those who had been diagnosed by a physician as photosensitive; (2) those who were currently taking medication or undergoing outpatient treatment for a serious illness; (3) those who were currently under medical supervision for exercise or diet therapy; (4) those who might be allergic to any of the ingredients (yeast extract) in the test food; (5) those who had a current or past history of substance use disorder; (6) those who were currently being hospitalized for mental disorders (depression, etc.) or sleep disorders (insomnia, sleep apnea, etc.) or who had a history of mental illness in the past; (7) those who had an irregular schedule due to night work, shift work, and so on; (8) those with extremely irregular eating, sleeping, or other lifestyle habits; (9) those who have an extremely unbalanced diet; (10) those with a serious current or past disease such as brain disease, malignant tumor, immunological disease, diabetes, liver disease (hepatitis), kidney disease, heart disease, thyroid disease, adrenal gland disease, or other metabolic diseases; (11) those who have continuously used or taken anti-inflammatory medications (topical or oral) for the skin on the UV-exposed area (back) more than once per month; (12) those who have factors on the skin of the UV-exposed area (back) that may affect the results of the study (diseases such as atopic dermatitis or urticaria, inflammation, eczema, trauma, acne, pimples, warts, moles, tattoos, or traces of these); (13) those who have used drugs, quasi-drugs, cosmetics, health foods, dietary supplements, and other application preparations or foods containing the following ingredients: glutathione, cysteine, yeast extract, vitamin C, vitamin E, vitamin A (retinol), tretinoin, placenta, hyaluronic acid, collagen, astaxanthin, xanthophyll, lycopene, beta-carotene, procyanidin, collagen peptide, heparin-like substances, and so on that emphasize whitening effects, sunburn prevention effects, or skin quality improvement (effects of preventing or improving blemishes, preventing or improving rough skin, or promoting turnover), either orally or topically on the UV-exposed area (back) within 3 months from the date of obtaining consent, and who are unable to refrain from use or ingestion during the study period; (14) those who have been exposed to UV rays beyond their daily activities, such as prolonged outdoor work, sports, swimming in the sea, tanning salons, and so on, in the 2 months prior to the SCR test; (15) those who were currently using esthetic salons, cosmetic treatments (e.g., laser treatment), or cosmetic treatments (e.g., ZeoSkin) on the UV-exposed area (back) under the supervision of a physician; (16) those who intended to undergo hair removal on the UV-exposed area (back) during the study period; (17) those who had participated in another clinical trial (research) within 3 months prior to the date of consent, or who plans to participate in another clinical trial (research) during the study period; (18) those who were currently pregnant or lactating, or who may become pregnant or begin lactating during the study period; (19) those who had difficulty in completing various questionnaires; (20) those who were judged to be an unsuitable subject based on clinical laboratory values and measurements at the time of SCR; and (21) any other participant who was judged to be an unsuitable by the investigator.

### Intervention

Participants in the cysteine peptides group received the test food, consisting of 325 mg/3 tablets/day as HITHION YH-15, which contained 48 mg/3 tablets/day of cysteine peptides, determined by a modified high-performance liquid chromatographic (HPLC) method using Ellman’s reagent (5,5′-dithiobis(2-nitrobenzoic acid), DTNB)^14^ and dithiothreitol (DTT), a reducing agent, for GSSG^15,16^. Participants in the placebo group consumed the placebo food in which yeast extract was replaced with maltitol, a major component of the tablet.

### Evaluation of UV-induced erythema and pigmentation

Changes in UV-B-induced erythema (MED) and pigmentation were compared between the groups. MED was determined by irradiation with 12 levels of UV-B at 290–320 nm (11.57, 13.30, 15.30, 17.56, 20.22, 23.28, 26.73, 30.72, 35.38, 40.70, 46.82, and 53.87 mJ/cm^2^) before and after 4 weeks of intervention, using a solar simulator (601–300 V2.5 UV Multiport: Solar Light Company, Inc., Glenside, PA, USA). Pigmentation was determined by measuring the L* value and melanin index 7 days after irradiation at 1.5 MED, based on the MED before the intervention, using a CM-26d spectrophotometer (Konica Minolta Japan, Inc., Japan) and MEXAMETER MX 18 (Courage + Khazaka electronic GmbH, Cologne, Germany), respectively. Each pigmentation parameter was measured before and after 5 weeks of intervention and corrected as ΔL* and Δmelanin, where Δ = 1.5 MED irradiated area—non-irradiated area. All irradiations and measurements were performed on the backs of the participants. The changes before and after the intervention were calculated for statistical comparison.

### Safety evaluation

During the study period, adverse events were evaluated using a Daily Life Survey. The investigator was consulted in cases of new adverse events or abnormal variations.

### Statistical analysis

Data are presented as mean ± SD for baseline characteristics of participants and mean ± standard error for outcomes. Population variation was first checked for normality using the Shapiro–Wilk test. Based on the results of the tests, a nonparametric test was performed for the MED; an F-test was performed for the L* value and melanin indices, and an appropriate parametric test was performed based on the test results. Differences between groups in MED were assessed by the Wilcoxon rank-sum test and those in ΔL* value and Δmelanin index by Student’s unpaired t-test. Within-group differences in MED were assessed using the Wilcoxon signed-rank test and those in ΔL* value and Δmelanin index using Student’s paired t-test. Probabilities of < 5% (*, *p* < 0.05; **, *p* < 0.01; ***, *p* < 0.001) were considered statistically significant. IBM SPSS STATISTICS 25 (IBM Corp, Armonk, NY, USA) was used to perform the data analysis.

### Institutional review board

The study was conducted in accordance with the Declaration of Helsinki and approved by the Ethics Review Committee of Youga Allergy Clinic Clinical Research (IRB # 21000023; Approved 20/1/2023). The study was registered in the UMIN Clinical Trials Registry on 27/1/2023 (UMIN000050157).

### Informed consent

Informed consent was obtained from all subjects involved in the study.

## Results

### Participants

**Fig. 1:**
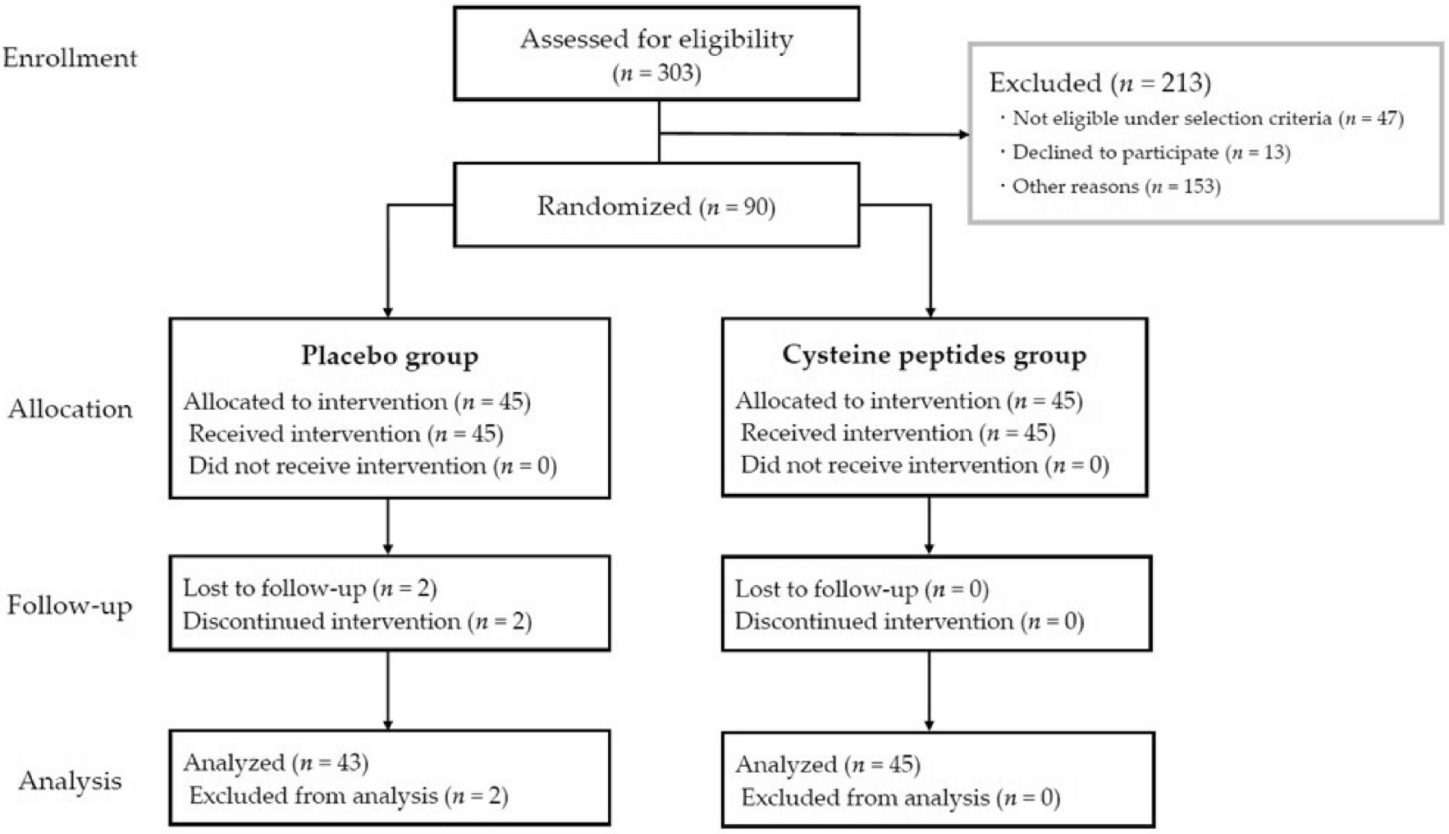
Consolidated standards of reporting trials (CONSORT) diagram.

A total of 303 participants were screened, of which 90 participants (30–59 years old, 56 females and 34 males) were enrolled and randomized into two groups: cysteine peptides (*n* = 45) and placebo (*n* = 45). No significant differences were observed in the baseline characteristics of the study participants regarding age, sex, skin phototypes, MED, L* value, and melanin indices 7 days after 1.5 MED irradiation between the placebo and cysteine peptides groups (Table 2). Two participants in the placebo group discontinued the study, one, because of a cold, and the other, for personal reasons. Finally, the data of 88 participants (placebo group, *n* = 43; cysteine peptides *n* = 45) were analyzed using per-protocol set analysis **(**Fig. 1), where all baseline characteristics of the analyzed participants remained non-significant between the groups (Table S1). All participants had an intake rate of > 97.40%. There was no statistically significant difference in ingestion rate between groups (99.94 ± 0.39% and 100.00 ± 0.00% in the cysteine peptides and placebo groups, respectively). In addition, vital signs such as systolic and diastolic blood pressures and physical measurements such as body weight and BMI at pre- and post-intake also remained non-significant between groups and within groups, except for pulse in the placebo group, which significantly decreased after the study period, however, there was no causal relationship with the test food (Table S2).

**Table 2:** Baseline characteristics of study participants (*, *p* < 0.05; **, *p* < 0.01; ***, *p* < 0.001).

	Placebo (n = 45)	Cysteine peptides (n = 45)	*p* value
Age (mean ± SD)	47.5 ± 5.6	47.5 ± 7.8	1.000
Female, n (%)	29 (64%)	27 (60%)	0.827
Skin phototypes (II, III)	2.3 ± 0.4	2.3 ± 0.5	0.816
MED (mJ/cm^2^) (mean ± SD)	33.8 ± 4.1	33.9 ± 4.8	0.919
L*value (mean ± SD) 7 days after 1.5 MED irradiation	60.9 ± 2.7	61.3 ± 2.4	0.415
Melanin indices (mean ± SD) 7 days after 1.5 MED irradiation	151.8 ± 45.6	148.6 ± 39.7	0.720

### Effects of cysteine peptides on UV-B-induced erythema and pigmentation

**Fig. 2:**
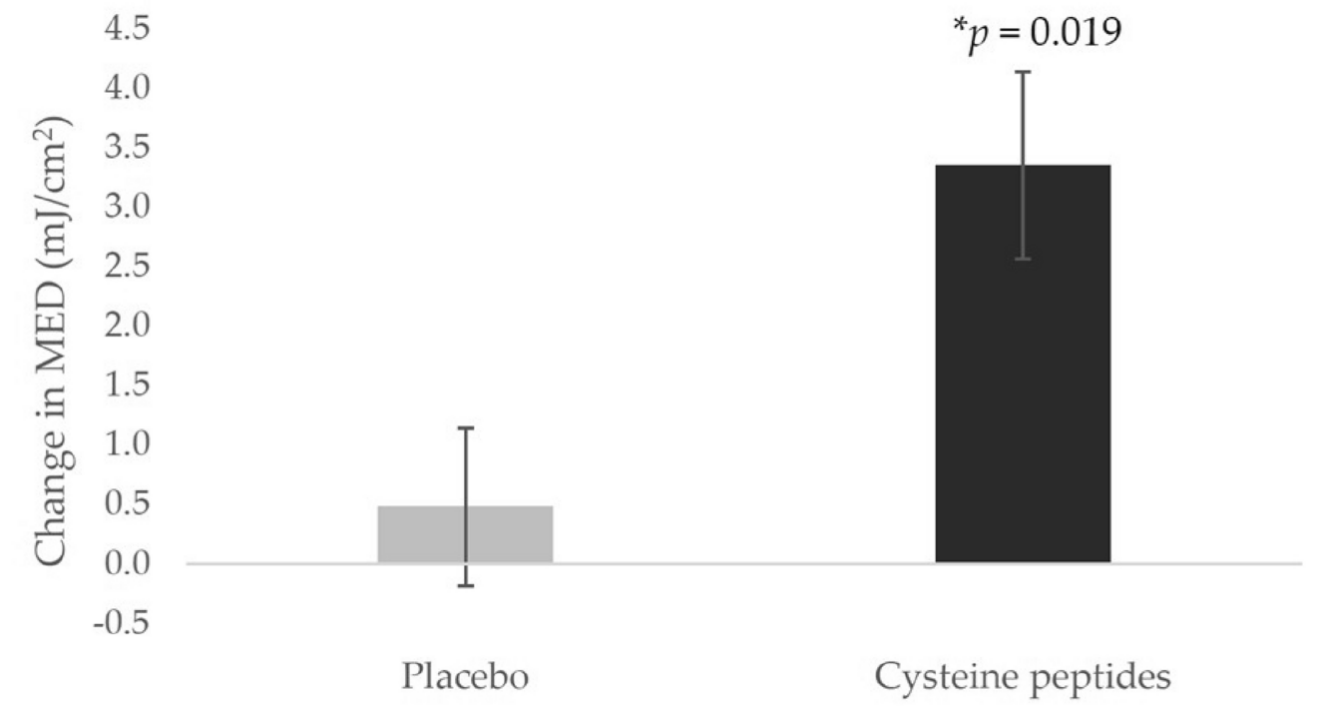
Four-week oral supplementation of cysteine peptides increased minimal erythema dose (MED). Changes from baseline in MED in the placebo group (gray) and cysteine peptides group (black) are indicated. *, *p* < 0.05; **, *p* < 0.01; ***, *p* < 0.001 using the Wilcoxon rank-sum test. Error bars indicate the standard error.

**Fig. 3:**
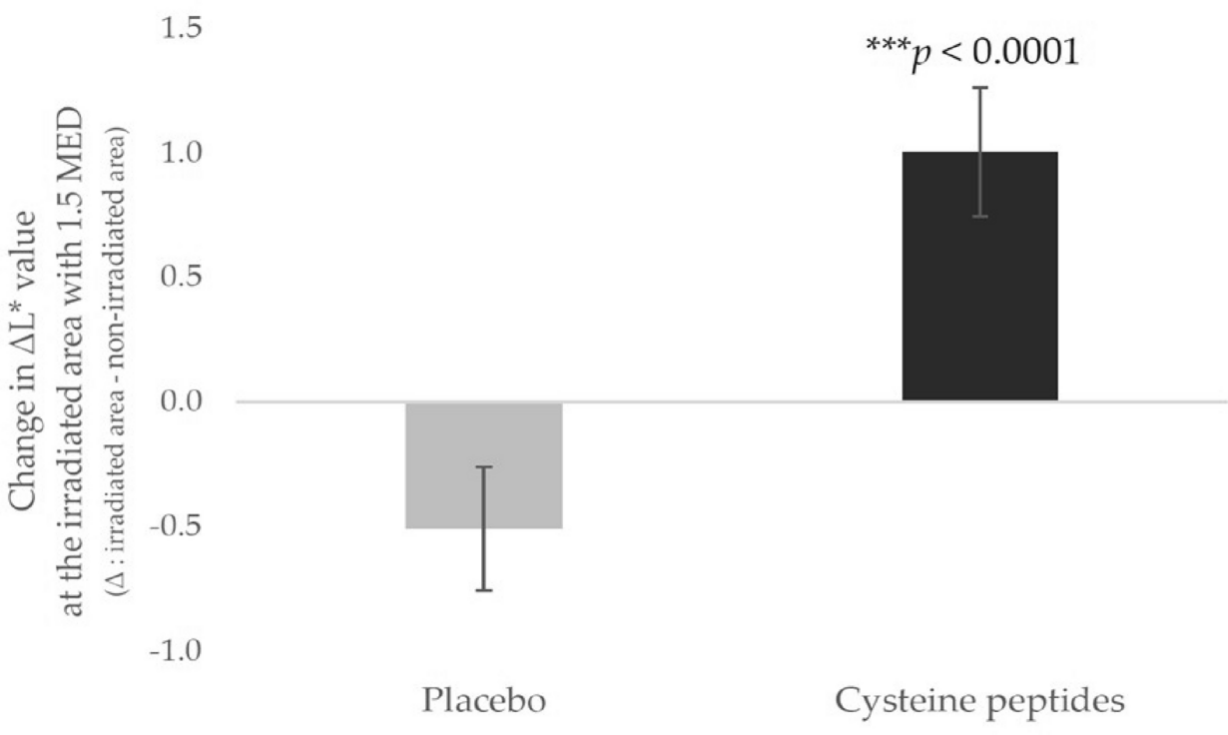
Five-week oral supplementation of cysteine peptides suppressed the pigmentation at the UV-B irradiated area. ΔL*value was calculated as the difference between irradiated area and non-irradiated area. Changes from baseline in ΔL*value at the irradiated area 7 days after irradiation with 1.5 MED in the placebo group (gray) and cysteine peptides group (black) are indicated. *, *p* < 0.05; **, *p* < 0.01; ***, *p* < 0.001 using the unpaired t-test. Error bars indicate the standard error.

The change in MED significantly increased (*p = 0.019 by the Wilcoxon rank-sum test, interquartile range from the 25th to 75th percentile of the change in MED = 0.0–5.3) in the cysteine peptides group (3.3 ± 0.8 mJ/cm^2^) compared to that in the placebo group (0.5 ± 0.7 mJ/cm^2^), indicating that oral administration of cysteine peptides suppressed UV-B-induced erythema (Fig. 2). The change in ΔL* value significantly increased (***p < 0.0001 using Student’s unpaired t-test, 95% Confidence Interval (CI) = 0.8–2.2) in the cysteine peptides group (1.0 ± 0.3) compared to that in the placebo group (− 0.5 ± 0.2), thus indicating pigmentation suppression (Fig. 3). The changes in L* values at the non-irradiated area over 5 weeks were significantly different in both groups, but in the opposite manner. The change in L* value in the cysteine peptides group was significantly increased (*p = 0.0332 by Student’s paired t-test), indicating that the skin became brighter after 5 weeks of cysteine peptides intake. The L* value in the placebo group was significantly decreased (*p = 0.0197 by Student’s paired t-test), indicating that the skin became darker after 5 weeks of placebo intake. (Fig. S1) The change in Δmelanin index was not significantly different (*p* = 0.144 using Student’s unpaired t-test, 95% CI = -19.2 to 2.9) between the groups (cysteine peptides group: −11.4 ± 4.3; placebo group: − 3.2 ± 3.4) due to large individual differences (Fig. S2). The Δmelanin indices significantly decreased after 5 weeks of intervention only in the cysteine peptides group (*p = 0.011 by Student’s paired t-test), compared to the baseline (Fig. S3). The change in melanin indices at the non-irradiated area in the cysteine peptides group was not significantly different although that in the placebo group significantly increased (*p = 0.0403 by Student’s paired t-test). (Fig. S4).

### Clinical safety

No adverse events related to the administration of cysteine peptides were observed during the study period.

## Discussion

Various studies on the effects of GSH on skin properties have been conducted using an effective amount of 250 or 500 mg per day^6–8^. However, this study, together with our previous study^12^, showed conclusively that a low dose of cysteine peptides (48 mg/day) is significantly effective in suppressing UV-B-induced skin damage and brightening the skin. These findings of dose independence may provide new insights into the association between the oral intake of GSH and cellular GSH levels. Some human studies have demonstrated that even relatively large doses (1,000 or 3,000 mg per day) of oral GSH supplementation do not increase blood GSH levels^17,18^. A previous animal study with rats also suggested that oral administration of GSH did not significantly affect GSH levels in the liver when sufficient amounts of GSH can be synthesized^19^. By contrast, a 6-month-long human study utilizing dosages of 250 or 1,000 mg/day led to significant increases in body stores of GSH in a dose- and time-dependent manner when the possible differences in erythrocyte volume and number, which significantly influence GSH levels, were taken into consideration^20^. Furthermore, levels of protein-bound forms of GSH, γ-Glu-Cys, and Cys-Gly in plasma, were found to significantly increase after oral GSH administration (50 mg/kg body weight)^21^. This may be one of the reasons why oral administration of free GSH did not effectively increase blood GSH levels. Based on these previous human studies, the effect of oral supplementation of GSH on GSH levels in the body remains inconclusive because of the complex disposition of oral GSH in the body and the handling and measurement techniques required to properly quantify GSH levels. By contrast, in vitro studies have shown that GSH depletion leads to an increased susceptibility to oxidative damage and increased ROS levels, eventually resulting in cell death^22,23^. GSH depletion is also involved in numerous diseases through chronic proinflammatory conditions in humans^24^. These reports suggest that the effects of oral supplementation with GSH may not be dose-dependent but are more dependent on the baseline characteristics of the subjects. To design the present clinical study, we hypothesized several characteristics of healthy subjects who are more susceptible to UV-B-induced skin damage and may receive more benefits from the cysteine peptides supplementation and implemented them in the inclusion criteria. The first characteristic was a lower MED and the skin phototype. A previous study reported that for subjects with a lower MED (25–30 mJ/cm^2^), formation of cyclobutane pyrimidine dimer, a major type of UV-B induced DNA damage, at post-UV irradiation was higher^25^. In addition, subjects with skin phototype I tend to form more cyclobutane pyrimidine dimers than those with skin phototype III^26^. To test participants at a relatively higher risk of UV-B-induced skin damage, who have relatively lighter skin tones, we included participants with a lower MED among those with skin phototypes II or III. The second characteristic is advanced age. GSH in the body decreases with age in people older than 30 years^27^; hence, we included individuals aged 30–59 years. Furthermore, many studies on skin properties have been conducted in females because of the difficulty of including male participants in similar studies. In this study, we aimed to ensure that at least 30% of the participants were males, so that the male-to-female ratio of the study population would be more representative of the national population. Unfortunately, the cellular GSH level was not measured in this study, which limits the discussion of the association between oral administration of cysteine peptides and cellular GSH levels. However, the significant effect of the low dose of cysteine peptides on UV-B-induced erythema and pigmentation shown in this study suggests that this is largely due to the characteristics of subjects who were susceptible to UV-B-induced skin damage and therefore benefited from the cysteine peptides supplementation.

GSH is one of the non-enzymatic antioxidants in skin cells that exists in a concentration gradient and is more abundant in the outer layer^28^. They play crucial roles in maintaining an optimal redox balance by quenching ROS and protecting against oxidative stress and UV-B-induced skin deterioration^2,29^. A negative correlation between GSH levels and DNA damage has been observed in the liver and kidney cells of aging mice^30^. GSH is potentially involved in DNA repair and multiple cell signaling pathways^31^. GSH and γ-Glu-Cys are known to play important roles in the endogenous antioxidant defense system against UV-B-induced oxidative stress^32^. Several in vitro and in vivo studies investigated the protective effect of γ-Glu-Cys against UV-B radiation demonstrating both its antioxidant and anti-inflammatory properties^33–35^. Inflammatory stimuli such as UV-B irradiation increases the expression of glutathione synthetase through activation of nuclear factor-erythroid 2-related factor (Nrf2) and nuclear factor kappa B (NF-κB) pathways, thereby stimulating GSH synthesis from γ-Glu-Cys^33^. Unlike GSH, γ-Glu-Cys can be easily taken up by cells and suppresses excess ROS accumulation and GSH depletion by increasing GSH levels^33^. An in vitro study of pretreatment with γ-Glu-Cys demonstrated its effects of directly restoring the antioxidant defense system after exposure to UV-B radiation, reducing apoptosis rate, preventing DNA damage, and suppressing the activation of the mitogen-activated protein kinase (MAPK) pathways^34^. Based on these previous findings, GSH and its constituents are expected to play vital roles in protecting cells from UV through the antioxidant defense by eliminating ROS and direct protection and repair of cells and DNA. No human studies have yet investigated the protective effects of oral supplementation with GSH or its constituents against UV-B-induced skin damage, although many studies have demonstrated the skin-brightening effect of oral GSH intake.

The antioxidant ability of the cysteine peptides was considered the primary factor in the suppression of UV-B-induced erythema and pigmentation in this study. We determined the radical scavenging rate of the yeast extract HITHION YH-15 compared to GSH alone using the 1,1-diphenyl-2-picrylhydrazyl (DPPH) radical scavenging assay, the most commonly used method to determine antioxidant ability^35^. Our results indicated that HITHION YH-15 (2.4 mg/ml, which includes 0.3 mg/ml GSH) has significantly greater radical scavenging capacity (*p = 0.042; Student’s unpaired t-test) compared to the equivalent amount of GSH (0.3 mg/ml; Fig. S5), suggesting that the other constituents of cysteine peptides, including γ-Glu-Cys and Cys-Gly, may also contribute to its antioxidant ability. However, this in chemico assay does not reflect the oral intake of cysteine peptides because its degradation and resynthesis in the body have not been considered^36^. Orally-ingested GSH is directly absorbed only in small amounts in the intestines via the GSH transporter and then transported to the blood plasma in bound forms with proteins, including albumin and low-molecular-weight thiol compounds^37^. The majority of orally taken GSH is degraded extracellularly by degradative enzymes such as γ-glutamyl transpeptidase and dipeptidase into dipeptides and amino acids such as cysteine, glutamyl acid, and glycine. These amino acids are then taken up by cells via amino acid transporters and intracellularly regenerate GSH by γ-glutamyl-cysteine synthetase and glutathione synthetase^38,39^. Oral administration of GSH precursors such as N-acetylcysteine (NAC) and glycine is beneficial for increasing cellular GSH levels in patients with GSH deficiency^24^. γ-Glu-Cys was also evaluated to be more effective in elevating cellular GSH levels under lipopolysaccharide stimulation and exhibit a stronger anti-inflammatory effect than NAC in vitro^33^. The major rate-limiting factor in GSH synthesis in cells is the amount of cysteine present^40^. Glycine is also considered a rate-limiting factor in GSH production, and human studies have suggested that oral administration of glycine potentially counteracts oxidative stress and inflammation^41,42^. Therefore, dipeptides such as γ-Glu-Cys and Cys-Gly in cysteine peptides can be additional sources of cysteine as well as glutamyl acid and glycine to enhance intracellular GSH production when GSH is depleted due to UV-B-induced oxidative stress.

The significant suppression of UV-induced pigmentation as ΔL* value by cysteine peptides demonstrated in this study can be mainly explained by the multiple inhibitory mechanisms of melanin production by GSH such as disruption of the intracellular trafficking of tyrosinase, an enzyme necessary for melanin production, to melanosomes^43^, suppressing the activity of tyrosinase, and inducing the production of pheomelanin (light-colored melanin) instead of eumelanin (dark-colored melanin)^6,7^. As GSSG has been shown to reduce melanin indices and UV spots in a previous human study^7^, GSSG in cysteine peptides may contribute to the suppression of pigmentation through its reduction to GSH. In the present study, although the change in ΔL* showed a significant decrease, only the change in Δmelanin index did not show a significant difference between groups. We speculate that the main reason for not showing a significant difference is that the MEXAMETER used to measure the melanin index in this study cannot distinguish between the two types of melanin, eumelanin and pheomelanin, resulting in greater individual differences and variability. However, the cysteine peptide group only showed significantly lower levels at 5 weeks post intake compared to pre intake, suggesting that the cysteine peptides can reduce melanin production, although the effects may vary depending on the pigmentation patterns of the subjects. Further studies may be beneficial to investigate the individual variability of pigmentation, such as the production of eumelanin or pheomelanin more than the other, or the inclusion of other color indices, a* value representing redness, etc.

The significant decrease in L* values and increase in melanin indices in the non-irradiation area over 5 week period in the placebo group indicated that suntan can easily occur daily. However, the significant increase in L* values and the non-significant change in melanin indices non-irradiation area over 5 week period in the cysteine peptides group indicated that the daily intake of cysteine peptides may prevent pigmentation and brighten the skin itself. The skin-brightening effect of cysteine peptides shown in a previous study^12^ is probably due to the suppression of extra pigmentation from daily UV exposure and the promotion of skin turnover via the regulation of other antioxidants such as vitamin C^44^.

This study has several limitations. Since levels of cellular GSH or its protein-bound forms in plasma were not measured, there was a limited focus on the associations among the oral supplementation of low-dose cysteine peptides, changes in GSH levels, and UV protective effects. Further in vivo studies are required to measure the changes in cellular and epidermal GSH concentrations due to the oral administration of the cysteine peptides, with labeling of the four components of the cysteine peptides to investigate their dispositions. Because the effect of GSH alone was not tested in this study for comparison, another clinical trial investigating the effects of GSH alone is needed to confirm the necessity of the cysteine peptides. A low dose of cysteine peptides may be involved in multiple mechanisms of skin health promotion, but the precise mechanisms or new insights to explain its effects were not demonstrated in this study. This study was conducted in Japan, and the number and characteristics of participants were therefore limited. Moreover, a larger number of subjects with diverse characteristics including other skin types such as IV or V must be included in future studies to confirm and generalize this effect. In addition, this study used only UV-B irradiation, which is not equivalent to the UV radiation to which we are exposed on a daily basis. Further studies coupled with studies on the effects of UV-A irradiation help assess the long-term effect of cysteine peptides on photoaging.

Oral administration of cysteine peptides, as a nutricosmetic^45^, may not only be beneficial for maintaining skin health but also for contributing to individual overall health and well-being by encouraging people to have moderate sun exposure and improving vitamin D status^46^. Recently, 98% of Japanese individuals have been found to have vitamin D deficiency^47^. Lifestyle changes, including the excessive use of physical UV protection to avoid sunburn and suntan, result in inadequate exposure to sunlight. However, UV-B radiation of the skin is also essential for the effective and rapid production of vitamin D, which attenuates premature skin aging and cancer by inducing antioxidant responses, inhibiting DNA damage, and inducing DNA repair mechanisms^48,49^. In addition, vitamin D has been reported to upregulate cellular GSH levels in vitro by activating glutamate cysteine ligase and glutathione reductase, and decreasing ROS and proinflammatory cytokines^50^. Besides external UV care, such as sunscreen, options for internal care with oral supplementation of cysteine peptides may encourage people to partake in moderate sun exposure, allowing them to benefit from the positive consequences of vitamin D and GSH production while minimizing UV-B-induced skin damage, thereby promoting individual well-being.

In conclusion, the present study demonstrated that oral administration of the low dose of cysteine peptides (48 mg/day) suppresses UV-B-induced erythema and pigmentation, possibly through multiple mechanisms. Thus, cysteine peptides have great potential as a nutricosmetic to maintain skin health and well-being.

## Supplementary Information

Supplementary Information.

## Data Availability

The original contributions presented in the study are included in the article/supplementary material, further inquiries can be directed to the corresponding author.
