# Supplementary material for: Oral administration of cysteine peptides attenuates UV-B-induced skin erythema and pigmentation in humans
Source: Sci Rep. 2024 Sep 27;14:22163. doi: 10.1038/s41598-024-73447-z (PMC11436644; doi:10.1038/s41598-024-73447-z)
Supplement: Supplementary file 1 — Supplementary Information. [file 41598_2024_73447_MOESM1_ESM.pdf]

Table S1. Baseline characteristics of analyzed participants (*, $p < 0.05$; **, $p < 0.01$; ***, $p < 0.001$)

	Placebo (n = 43)	Cysteine peptides (n = 45)	*p* value
Age (mean±SD)	47.5±5.8	47.5±7.8	0.987
Female, n (%)	27 (63%)	27 (60%)	0.791
Skin photo types (II, III)	2.3±0.4	2.3±0.5	0.731
MED (mJ/cm^2) (mean±SD)	33.8±4.2	33.9±4.8	0.566
L*value (mean±SD)<br>7days after 1.5MED irradiation	60.8±2.7	61.3±2.4	0.32
Melanin indices (mean±SD)<br>7days after 1.5MED irradiation	152.3±46.6	148.6±39.7	0.686

Table S2. Vital signs and physical measurements at pre- and post-intake (*, $p < 0.05$)

	Measurement point	Group	Number of cases	Average ± SD	*p* value Between groups	*p* value Within a group
Systolic blood pressure (mmHg)	Pre-intake	Cysteine peptides	45	125.4 ± 12.6	0.769	-
		Placebo	45	126.2 ± 12.5		-
	Post-intake	Cysteine peptides	45	126.6 ± 11.9	0.267	0.313
		Placebo	43	123.7 ± 12.7		0.068
Diastolic blood pressure (mmHg)	Pre-intake	Cysteine peptides	45	81.4 ± 9.0	0.875	-
		Placebo	45	81.1 ± 9.8		-
	Post-intake	Cysteine peptides	45	81.8 ± 8.1	0.422	0.632
		Placebo	43	80.3 ± 9.3		0.393
Pulse (bpm)	Pre-intake	Cysteine peptides	45	72.8 ± 9.5	0.313	-
		Placebo	45	74.8 ± 9.5		-
	Post-intake	Cysteine peptides	45	70.8 ± 9.3	0.718	0.076
		Placebo	43	71.5 ± 9.8		* 0.010
Body weight (kg)	Pre-intake	Cysteine peptides	45	61.6 ± 11.2	0.292	-
		Placebo	45	59.2 ± 10.3		-
	Post-intake	Cysteine peptides	45	61.5 ± 11.2	0.353	0.521
		Placebo	43	59.3 ± 10.5		0.856
BMI (kg/m^2)	Pre-intake	Cysteine peptides	45	22.5 ± 3.0	0.812	-
		Placebo	45	22.4 ± 3.1		-
	Post-intake	Cysteine peptides	45	22.5 ± 3.0	0.773	0.620
		Placebo	43	22.3 ± 3.0		0.916

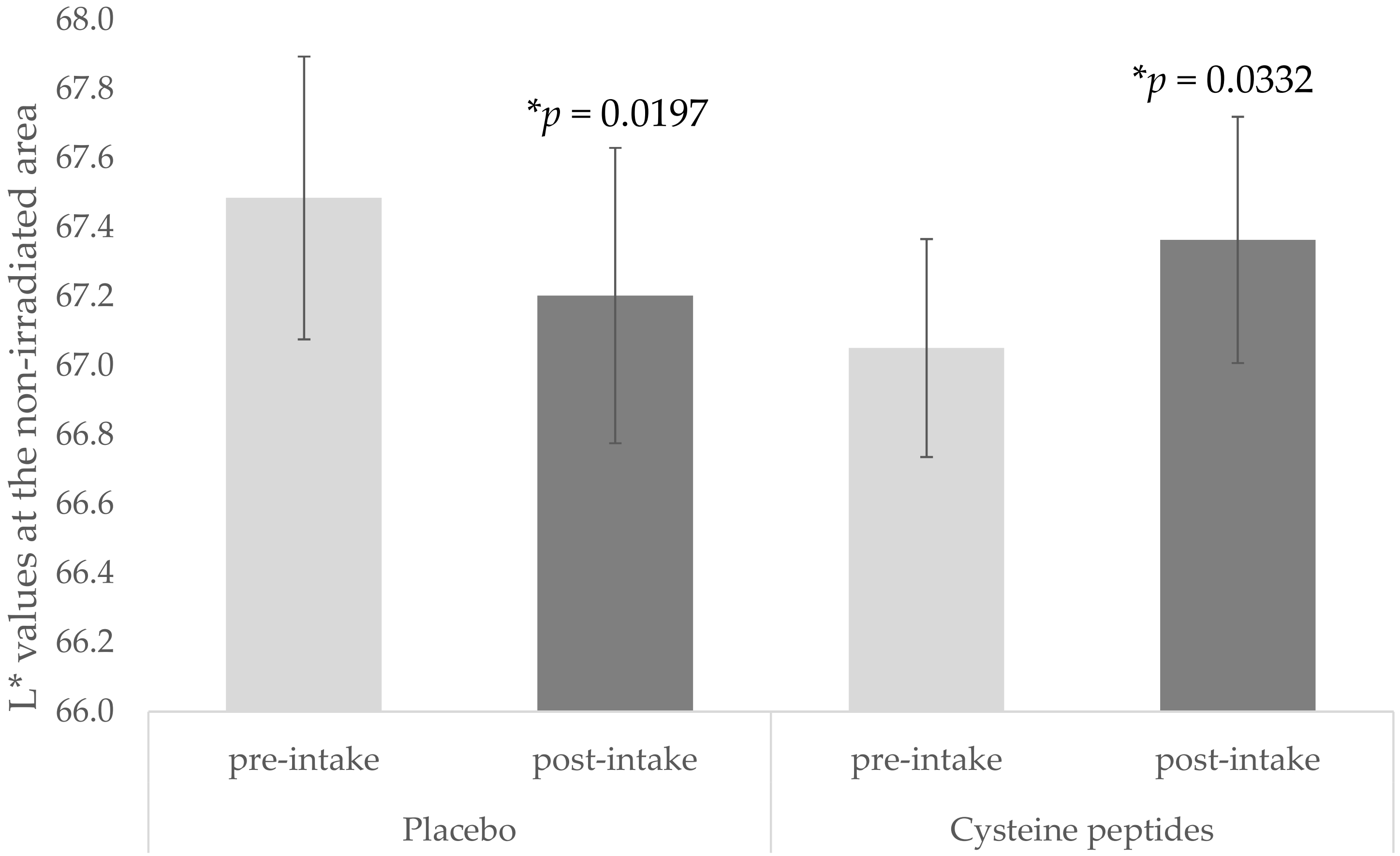

Figure S1. Changes in L* values at the non-irradiated area over 5 weeks were significantly different in both groups, but in the opposite manner. The change in L* value in the cysteine peptides group significantly increased (*$p = 0.0332$ by Student's paired t-test), indicating that the skin became brighter after 5 weeks of cysteine peptides intake. The L* value in the placebo group significantly decreased (*$p = 0.0197$ by Student's paired t-test), indicating that the skin became darker after 5 weeks of placebo intake.
*, $p < 0.05$; **, $p < 0.01$; ***, $p < 0.001$ by unpaired t- test. Error bars indicate the standard errors.

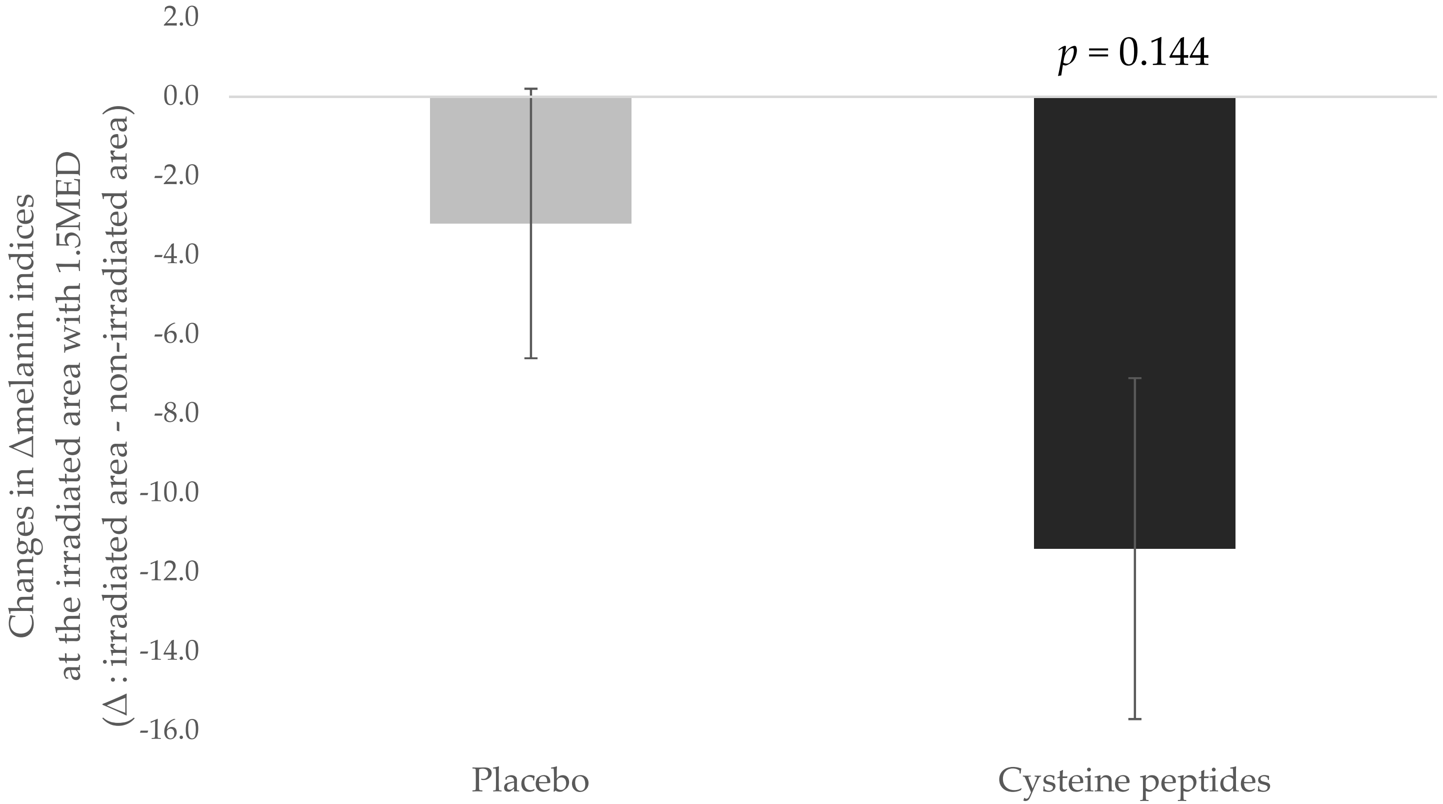

Figure S2. Five-week oral supplementation of cysteine peptides suppressed the pigmentation at the UV-B irradiated area, however, the changes in Δmelanin indices was not significantly different between the groups . Changes from baseline in Δmelanin index at 7 days after irradiation with 1.5MED in the placebo group (gray) and cysteine peptides group (black) were indicated. Δmelanin index was calculated by the difference between irradiated area and non-irradiated area. *, $p < 0.05$; **, $p < 0.01$; ***, $p < 0.001$ by unpaired t- test. Error bars indicate the standard errors.

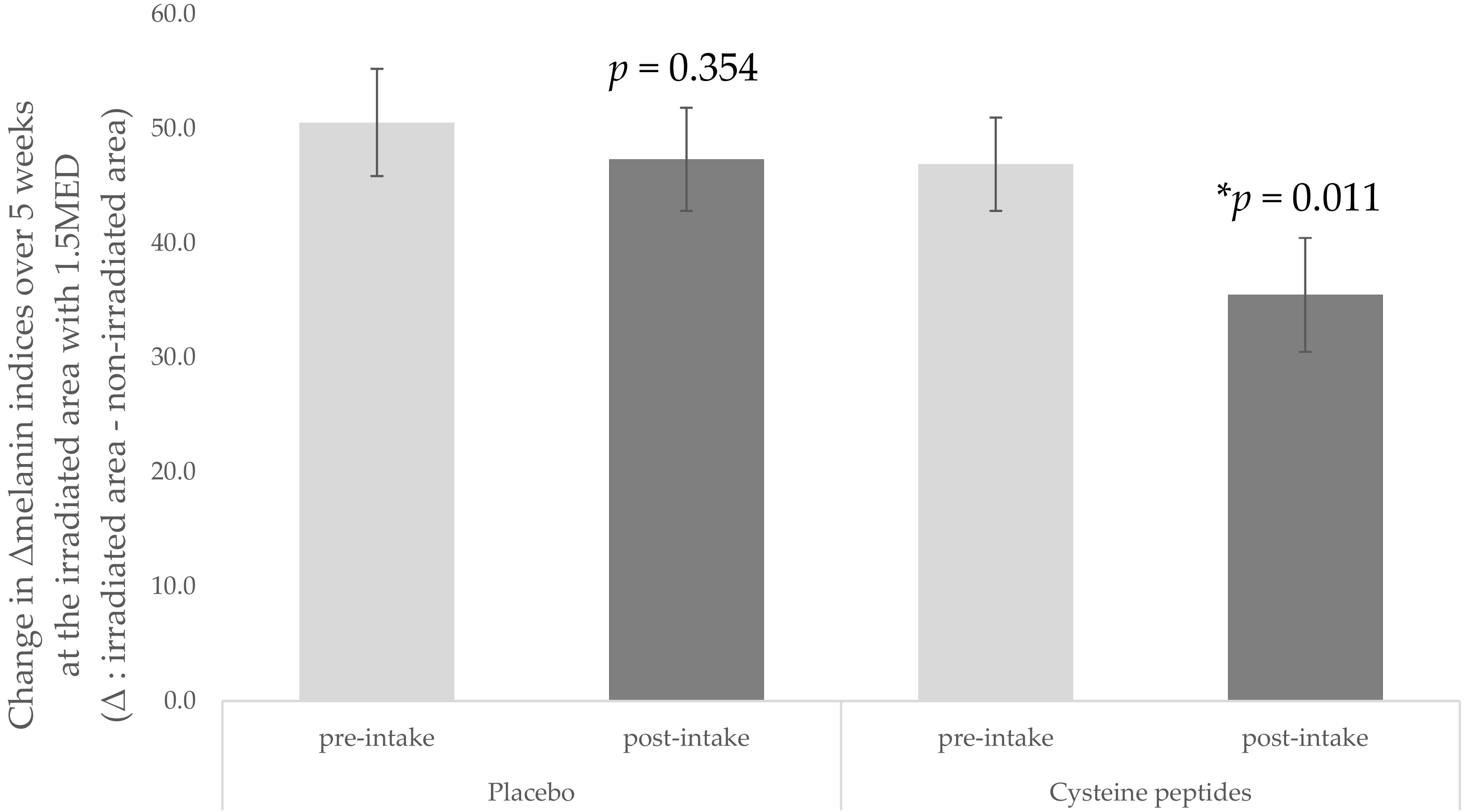

Figure S3. The Δmelanin indices at irradiated area with 1.5MED significantly decreased after 5 weeks of intervention in the cysteine peptides group (*$p = 0.011$ by Student's paired t-test), compared to the baseline, whereas that of placebo did not significantly changed.
*, $p < 0.05$; **, $p < 0.01$; ***, $p < 0.001$ by unpaired t- test. Error bars indicate the standard errors.

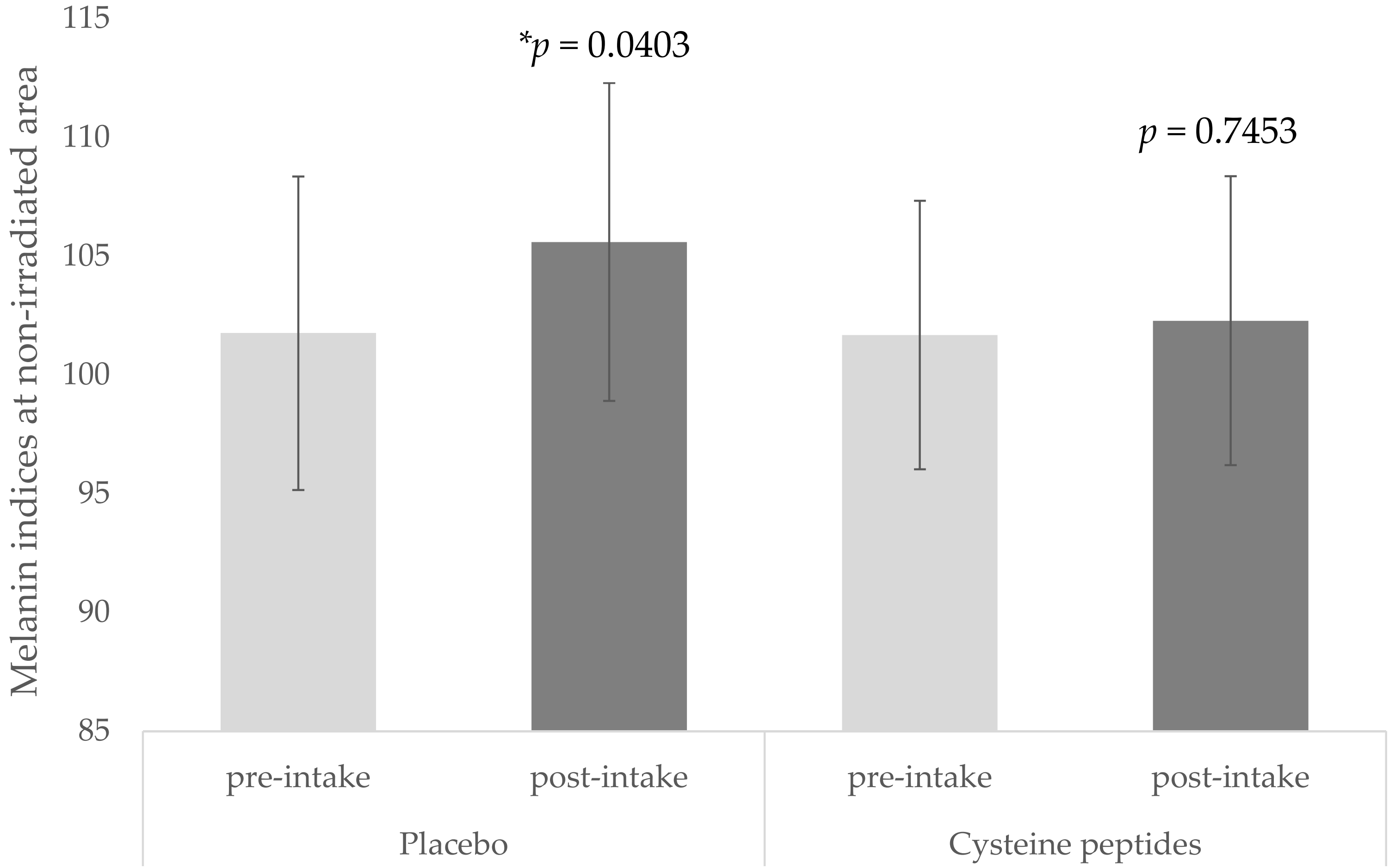

Figure S4. Changes in melanin indices at the non-irradiated area over 5 weeks in the cysteine peptides group was not significantly different although that in the placebo group significantly increased (*p = 0.0403 by Student's paired t-test). *, $p < 0.05$; **, $p < 0.01$; ***, $p < 0.001$ by unpaired t- test. Error bars indicate the standard errors.

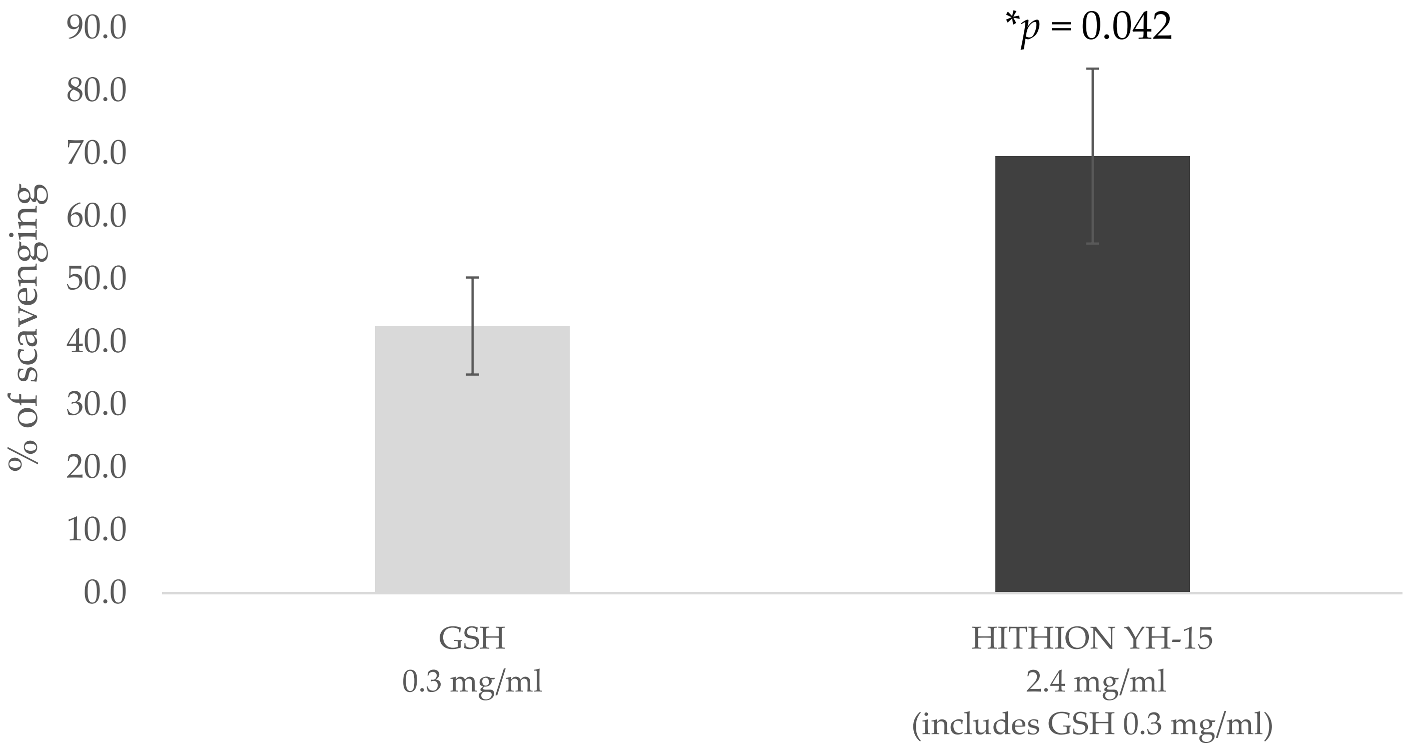

Figure S5. 1,1-diphenyl-2-picrylhydrazil (DPPH) radical scavenging activity (%) of GSH and HITHION YH-15 Radical scavenging activity in HITHION YH-15 (2.4 mg/ml including 0.3 mg GSH) showed the approximately 1.6 times higher than that in GSH (0.3 mg/ml).
*, $p < 0.05$; **, $p < 0.01$; ***, $p < 0.001$ by unpaired t- test. Error bars indicate the standard deviations.
